# Secure searching of biomarkers through hybrid homomorphic encryption scheme

**DOI:** 10.1186/s12920-017-0280-3

**Published:** 2017-07-26

**Authors:** Miran Kim, Yongsoo Song, Jung Hee Cheon

**Affiliations:** 1 Division of Biomedical Informatics, University of California- San Diego, San Diego, CA, 92093 USA; 2 Department of Mathematical Sciences, Seoul National University, GwanAkRo 1, Seoul, 08826 Republic of Korea

**Keywords:** Homomorphic encryption, Biomarkers

## Abstract

**Background:**

As genome sequencing technology develops rapidly, there has lately been an increasing need to keep genomic data secure even when stored in the cloud and still used for research. We are interested in designing a protocol for the secure outsourcing matching problem on encrypted data.

**Method:**

We propose an efficient method to securely search a matching position with the query data and extract some information at the position. After decryption, only a small amount of comparisons with the query information should be performed in plaintext state. We apply this method to find a set of biomarkers in encrypted genomes. The important feature of our method is to encode a genomic database as a single element of polynomial ring.

**Result:**

Since our method requires a single homomorphic multiplication of hybrid scheme for query computation, it has the advantage over the previous methods in parameter size, computation complexity, and communication cost. In particular, the extraction procedure not only prevents leakage of database information that has not been queried by user but also reduces the communication cost by half. We evaluate the performance of our method and verify that the computation on large-scale personal data can be securely and practically outsourced to a cloud environment during data analysis. It takes about 3.9 s to *search-and-extract* the reference and alternate sequences at the queried position in a database of size 4M.

**Conclusion:**

Our solution for finding a set of biomarkers in DNA sequences shows the progress of cryptographic techniques in terms of their capability can support real-world genome data analysis in a cloud environment.

## Background

The rapid development of genome sequencing technology enables us to access large genome dataset and it looks poised to make a significant breakthrough in medical research. While genomic data can be used for a wide range of applications including healthcare, biomedical research, and direct-to-consumer services, it has numerous special distinguishing features and it can violate personal privacy via genetic disclosure or genetic discrimination [1–3]. Due to these potential privacy issues, it should be managed with care.

There have been various privacy-enhancing techniques using cryptographic methods as outsourced analysis tools of genomic data. Recently, it has been suggested that we can preserve privacy through homomorphic encryption (HE), which allows computations to be carried out on ciphertexts. Yasuda et al. [4] gave a practical solution to find the location of a pattern in a text by computing multiple Hamming distance values on encrypted data. Lauter et al. [5] gave a solution to privately compute the basic genomic algorithms used in genome-wide association studies.

Homomorphic encryption can be applied to privacy-preserving sequence comparison, but it is still impractical for the analysis of entire human genome information. For example, Cheon et al. [6] presented a protocol to compute the edit distance on homomorphically encrypted data but it took about 27 s even on length 8 DNA sequence. It is not easy to efficiently approximate the edit distance over encryption even though the distance to a public human DNA sequence is given [7]. This inefficiency comes from the difficulty of homomorphic evaluation of equality test: Encrypting the inputs bit-wise and computing over the encrypted bits yield expensive computation cost (at least linear in the data bit-length).

In this paper, we suggest an efficient method to securely search a set of biomarkers using hybrid Ring-GSW homomorphic encryption scheme.

### Problem setting

The iDASH (Integrating Data for Analysis, ‘anonymization’ and SHaring) National Center organizes the iDASH Privacy & Security challenge for secure genome analysis. This paper is based on a submission to the task 3 in 2016 iDASH challenge: secure outsourcing of testing for genetic diseases on encrypted genomes. The goal of this task is to privately calculate the probability of genetic diseases through matching a set of biomarkers to encrypted genomes stored in a public cloud service. The requirement is that the entire matching process needs to be carried out using homomorphic encryption so that any information about database and query should not be revealed to the server during computation.

Suppose that the client has a Variation Call Format (VCF) file which contains genotype information such as chromosome number and position in the genome. It also contains some information for each position such as reference and alternate sequences, where each base must be one of SNPs: *A,T,G*, and *C*. The client encrypts the information using homomorphic encryption and the server calculates the exact match over the encrypted data. The outcome is the absence/presence of the specified biomarkers, that is, an encryption of 1 if matched; otherwise, an encryption of 0. Finally the client decrypts the result by the secret key of homomorphic encryption.

### Practical homomorphic encryption

Fully Homomorphic cryptosystems allow us to homomorphically evaluate any arithmetic circuit without decryption. However, the noise of the resulting ciphertext grows during homomorphic evaluations, slightly with addition but substantially with multiplication. For efficiency reasons, for tasks which are known in advance, we use a more practical *Somewhat Homomorphic Encryption* (SHE) scheme, which evaluates functions up to a certain complexity. In particular, two techniques are used for noise management of SHE: one is the *modulus-switching* technique introduced by Brakerski, Gentry and Vaikuntanathan [8], which scales down a ciphertext during every multiplication operation and reduces the noise by its scaling factor. The other is a *scale-invariant* technique proposed by Brakerski such that the same modulus is used throughout the evaluation process [9].

Let us denote by [ ·]_*Q*_ the reduction modulo *Q* into the interval $(-Q/2,Q/2]\cap \mathbb {Z}$ of the integer or integer polynomial (coefficient-wise). For a security parameter *λ*, we choose an integer *M*=*M*(*λ*) that defines the *M*-th cyclotomic polynomial *Φ*_*M*_(*X*). For a polynomial ring $\mathcal {R}=\mathbb {Z}[X]/ (\Phi _{M}(X))$, set the plaintext space to $\mathcal {R}_{t}:= \mathcal {R}/t\mathcal {R}$ for some fixed *t*≥2 and the ciphertext space to $\mathcal {R}_{Q}:= \mathcal {R}/Q\mathcal {R}$ for an integer *Q*=*Q*(*λ*). Let *χ*=*χ*(*λ*) denote a noise distribution over the ring *R*. We use the standard notation $a \leftarrow \mathcal {D}$ to denote that *a* is chosen from the distribution $\mathcal {D}$.

#### The basic scheme

The following is a description of basic homomorphic encryption scheme based on the hardness of (decisional) *Ring Learning with Errors* (*RLWE*) assumption, which was first introduced by Lyubashevsky et al. [10]. The assumption is that it is infeasible to distinguish the following two distributions. The first distribution consists of pairs (*a*_*i*_,*u*_*i*_), where *a*_*i*_ and *u*_*i*_ are drawn uniformly at random from $\mathcal {R}_{Q}$. The second distribution consists of pairs of the form (*a*_*i*_,*b*_*i*_)=(*a*_*i*_,*a*_*i*_ s+*e*_*i*_) where *a*_*i*_ is uniformly random in $\mathcal {R}_{Q}$ and s,*e*_*i*_ are drawn from the error distribution *χ*. To improve efficiency for HE, we use sparse secret keys s with coefficients sampled from {0,±1} as in [11].

- *RLWE.ParamsGen*(*λ*): Given the security parameter *λ*, choose an integer *M*, a modulus *Q*, a plaintext modulus *t* with *t*|*Q*, and discrete Gaussian distribution *χ*_*err*_. Output *params*←(*M,Q,t*,*χ*_*err*_).
- *RLWE.KeyGen*(*params*): On the input parameters, let *N*=*ϕ*(*M*) and choose a sparse random s from {0,±1}^*N*^. Generate an *RLWE* instance (*a,b*)=(*a*,[−*a* s+*e*]_*Q*_) for *e*←*χ*_*err*_. We set the secret key *sk*←s and the public key *pk*←(*a,b*).
- *RLWE.Enc*(*m*,*pk*): To encrypt $m \in \mathcal {R}_{t}$, choose a small polynomial *v* and two Gaussian polynomials *e*_0_,*e*_1_ over $\mathcal {R}$ and output the ciphertext
  $$\begin{array}{ll} \mathsf{ct} & \leftarrow(c_{0},c_{1})\\ & =((Q/t) m,0) + (bv+e_{0}, av+e_{1}) \in \mathcal{R}_{Q}^{2}. \end{array} $$
- *RLWE.Dec*(*ct*,*sk*): Given a ciphertext *ct*=(*c*_0_,*c*_1_), output *m*←⌊(*t*/*Q*)·[*c*_0_+s·*c*_1_]_*Q*_⌉.
- *RLWE.Add*(*ct*,*ct* ^′^): Given two ciphertexts *ct*=(*c*_0_,*c*_1_) and *ct* ^′^=(*c*0′,*c*1′), the homomorphic addition is computed by *ct*_*add*_←([*c*_0_+*c*0′]_*Q*_,[*c*_1_+*c*1′]_*Q*_).

Throughout this paper, we assume that the integer *M* is a power of two so that *N*=*M*/2 and *ϕ*_*M*_(*X*)=*X* ^*N*^+1. We adapt the conversion and modulus-switching techniques of [12]. The conversion algorithm changes an *RLWE* encryption of $m=\sum _{i} m_{i} X^{i}$ into an *LWE* encryption of its constant term *m*_0_, and the modulus switching reduces the ciphertext modulus *Q* down to *q* while preserving the message. We note that an *LWE* ciphertext is represented as a vector in $\mathbb {Z}_{q}$ for some modulus *q*, and the decryption procedure is done by an inner product of the ciphertext and the secret key vector.

- *RLWE.Conv*(*ct*): Given a ciphertext *ct*=(*c*_0_,*c*_1_) with $c_{0}=\sum _{i} c_{0,i}X^{i}$ and $c_{1}=\sum _{i} c_{1,i}X^{i}$, output the vector *ct* ^′^=(*c*_0,0_,*c*_1,0_,−*c*_1,*N*−1_,…,−*c*_1,1_).
- *LWE.ModSwitch*(*ct*): Given a ciphertext $\mathsf {ct}\in \mathbb {Z}_{Q}^{N+1}$, output the vector $\mathsf {ct}'\leftarrow \lfloor {(q/Q)\cdot \mathsf {ct}}\rceil \in \mathbb {Z}_{q}^{N+1}$.

An *RLWE* ciphertext *ct*=(*c*_0_,*c*_1_) has the decryption structure of the form *c*_0_+*c*_1_·s=(*Q*/*t*)·*m*+*e* and its constant term is

$$c_{0,0}+ c_{1,0}s_{0} - \sum_{i=1}^{N-1} c_{1,N-i} s_{i}=(Q/t)\cdot m_{0}+e_{0}. $$

It can be represented as an inner product of a vector (*c*_0,0_,*c*_1,0_,−*c*_1,*N*−1_,−*c*_1,*N*−2_,…,−*c*_1,1_) and the desired *LWE* secret key $\vec s=(1,s_{0},\dots,s_{N-1})$. Hence the output of the conversion algorithm can be seen as an *LWE* encryption of *m*_0_. It is also easy to check that if $\mathsf {ct}\in \mathbb {Z}_{Q}^{N+1}$ satisfies $\langle {\mathsf {ct}},{\vec s}\rangle =(Q/t)\cdot m+e \pmod {Q}$, then the output of *LWE.ModSwitch* algorithm satisfies $\langle {\mathsf {ct}'},{\vec s}\rangle =(q/t)\cdot m+e' \pmod q$ for some *e* ^′^≈(*q*/*Q*)·*e*. These techniques have been proposed for an efficient bootstrapping [12], but they will play totally different roles in our application. Finally an *LWE* ciphertext of modulus *q* can be decrypted by $\vec s$ as follows.

- *LWE.Dec*(*ct*,*sk*): Given a ciphertext $\mathsf {ct}\in \mathbb {Z}_{Q}^{N+1}$, output the value $m\leftarrow \lfloor {(t/q)\cdot [\langle {\mathsf {ct}},{\vec s}\rangle ]_{q}}\rceil $.

If $\langle {\mathsf {ct}},{\vec s}\rangle =(q/t)\cdot m+e \pmod q$ for some small enough *e*, it returns the correct message *m* modulo *t*. More precisely, the decryption procedure works if |*te*/*q*|<1/2.

#### The Ring-GSW scheme

Gentry et al. [13] suggested a fully homomorphic encryption based on the *LWE* problem, where the message is encrypted as an approximate eigenvalue of a ciphertext. Ducas and Micciancio [12] described its *RLWE* variant. The *RGSW* symmetric encryption scheme consists of the following algorithms.

- *RGSW.ParamsGen*(·),*RGSW.KeyGen*(·): Use the same parameter *params* and secret key s with the basic *RLWE* scheme. Additionally set the decomposition base *B*_g_ and exponent *d*_g_ satisfying ${B}_{\mathsf {g}}^{{d}_{\mathsf {g}}}\ge Q$.
- *RGSW.Enc*(*m*,*sk*): To encrypt $m \in \mathcal {R}_{t}$, pick a matrix $\mathbf {a}\in \mathcal {R}_{Q}^{2{{d}_{\mathsf {g}}}}$ uniformly at random, and $\mathbf {e} \in \mathcal {R}^{2{{d}_{\mathsf {g}}}} \simeq \mathbb {Z}^{2{{d}_{\mathsf {g}}} \cdot n}$ with discrete Gaussian distribution *χ* of parameter *ς*, and output the ciphertext
  $$\mathsf{CT} \leftarrow [\mathbf{b}, \mathbf{a}] +\, m \mathbf{G} \in \mathcal{R}_{Q}^{2{{d}_{\mathsf{g}}} \times 2} $$
  where **b**=−**a**·s+**e** and the gadget matrix $\mathbf {G}= \left (\mathbf {I} ~\Vert ~ {B}_{\mathsf {g}}\mathbf {I} ~\Vert ~ \ldots ~\Vert ~ {B}_{\mathsf {g}}^{{{d}_{\mathsf {g}}}-1}\mathbf {I}\right)^{T} \in \mathcal {R}_{Q}^{2{{d}_{\mathsf {g}}} \times 2}$ for 2×2 identity matrix **I**.

Let $\phantom {\dot {i}\!}\mathsf {WD}_{{B}_{\mathsf {g}}}(\cdot)$ be the decomposition with the base *B*_g_, where the dimension of input vector is multiplied by *d*_g_ through this algorithm. The *RGSW* encryption of *m* satisfies $\mathsf {CT} \cdot (1,\mathsf {s})= m\cdot \left (1,\mathsf {s},\dots,{B}_{\mathsf {g}}^{{{d}_{\mathsf {g}}}-1},{B}_{\mathsf {g}}^{{{d}_{\mathsf {g}}}-1}\mathsf {s}\right)+\mathsf {e}$. Roughly, *m* is an *approximate* eigenvalue of $\phantom {\dot {i}\!}\mathsf {WD}_{{B}_{\mathsf {g}}}(\mathsf {CT})$ with respect to the eigenvector $\left (1,\mathsf {s},\dots,{B}_{\mathsf {g}}^{{{d}_{\mathsf {g}}}-1},{B}_{\mathsf {g}}^{{{d}_{\mathsf {g}}}-1}\mathsf {s}\right)$. In [14], the hybrid multiplication between *RGSW* ciphertexts and *RLWE* ciphertexts has been defined as follows.

- *Hybrid.Mult*(*CT*,*ct*): Given an *RGSW* ciphertext $\mathsf {CT}\in \mathcal {R}_{Q}^{2{{d}_{\mathsf {g}}}\times 2}$ and an *RLWE* ciphertext $\mathsf {ct}\in \mathcal {R}_{Q}^{2}$ output the vector $\mathsf {ct}'\leftarrow \mathsf {CT}^{T}\cdot {\mathsf {WD}_{{B}_{\mathsf {g}}}}(\mathsf {ct})$.

If *CT* and *ct* are *RGSW* and *RLWE* encryptions of *m* and *m* ^′^, respectively, their multiplication *ct* ^′^ is a valid *RLWE* encryption of *m* *m* ^′^. For convenience, we will denote *Hybrid.Mult*(*CT*,*ct*) algorithm by $\boxdot $, *i.e.,* $(\mathsf {CT},\mathsf {ct})\in \mathcal {R}_{Q}^{2{{d}_{\mathsf {g}}}\times 2}\times \mathcal {R}_{Q}^{2}\mapsto \mathsf {CT}\boxdot \mathsf {ct}\in \mathcal {R}_{Q}^{2}$.

## Methods

### Privacy-preserving database searching and extraction

Let us consider a database of a set of *n* tuples. Each tuple consists of pairs (*d*_*i*_,*α*_*i*_) for *i*=1,…,*n*, where *d*_*i*_ denotes a *data-tag* in the domain $\{0,1,\dots,\mathcal {T}-1\}$ and *α*_*i*_ represents the corresponding *value attribute* in a plaintext space $\mathbb {Z}_{t}\backslash \{0\}$.Note that all the tags should be distinct from each other. For instance, in the case of personal information database, *α*_*i*_ may be the age of user whose identity number is *d*_*i*_.

Given a *query tag* *d* from a tag domain and a *query value* *α* from a plaintext space, the matching problem is to determine the existence of an index *i* such that (*d*,*α*)=(*d*_*i*_,*α*_*i*_). Now consider the following simplified search query: select *α*_*i*_ if there exists an index *i* such that *d*_*i*_=*d*; otherwise zero (⊥). The purpose of this section is to store the database and carry out this search query on the public cloud. The server should learn nothing from encrypted query and any information other than the final result should not be leaked to user. Throughout this work, we will use semi-honest (honest but curious) adversary model, which is a standard assumption for evaluation of homomorphic encryption.

Our main idea is the following encoding method of database suitable for the efficient computation of equality test and extraction:

$$\mathsf{DB}(X)=\sum_{i}\alpha_{i}X^{d_{i}}\in\mathbb{Z}_{t}[X]. $$

The user encrypts this polynomial with the *RLWE* public-key encryption scheme and stores the ciphertext *ct*_*DB*_ in the server. At the query phase, given a query tag *d*, the user encrypts the monomial *X* ^−*d*^ with the *RGSW* symmetric encryption scheme and sends the ciphertext *CT*_Q_ to the server. We assume that the *RGSW* encryption scheme has the same secret key *sk* as the one of *RLWE* encryption scheme.

Given two ciphertexts *CT*_Q_←*RGSW*.*Enc*(*X* ^−*d*^) and *ct*_*DB*_←*RLWE*.*Enc*(*DB*(*X*)), the server first performs their multiplication to obtain an ciphertext, denoted by $\mathsf {ct_{mult}} = \mathsf {CT}_{\mathsf {Q}} \boxdot \mathsf {ct}_{\mathsf {DB}}$. It follows from the previous section that c *t*_*mult*_ is a valid *RLWE* encryption of the polynomial

$$\mathsf{DB}(X) \cdot X^{-d}= \sum_{i} \alpha_{i} X^{d_{i}-d} \in \mathcal{R}_{t}. $$

Since we use the cyclotomic polynomial *ϕ*_*M*_(*X*)=*X* ^*N*^+1 of power-of-two degree, the polynomial ring $\mathcal {R}$ has the property *X* ^*N*^=−1. Thus, for any tag *d*, the constant term of the polynomial *DB*(*X*)·*X* ^−*d*^ is *α*_*i*_ if there is some index *i* satisfying *d*=*d*_*i*_, otherwise zero.

Now the server applies the *RLWE.Conv* algorithm on c *t*_*mult*_ to compute an *LWE* encryption *ct*_*conv*_ of this constant term. This conversion procedure not only prevents the leakage of information that has not been queried but also reduces the size of output ciphertext by half. In addition, the (optional) modulus-switching procedure can be considered to get a ciphertext c *t*_*res*_ with a smaller modulus size and reduce the communication cost. Finally the user decrypts this *LWE* ciphertext and gets the desired value *α*_*i*_ or zero (⊥). Algorithm 1 summarizes the procedure of secure *search-and-extraction*.

Our method can be modified to support a secure comparison of data values using a hash (one-way) function. If hashed values of *α*_*i*_ are used as polynomial coefficients, our method will return a hashed value of *α*_*i*_ to the user instead of *α*_*i*_. The user may check whether the resulting value and the hashed query value are the same or not without knowing information about database.



### Comparison with related work

Equality test has been traditionally considered difficult to perform on homomorphic encryption, because of its large circuit depth [7, 15, 16]. They evaluate the equality test on each encrypted tuple of database, so at least *Ω*(*n*) homomorphic operations are required for searching on database of size *n*. In addition, Boneh et al. [17] does not protect the database information to the users, that is, the whole database can be recovered by the resulting ciphertext of a query. However, our method is very efficient in parameter size and complexity since it requires only a single hybrid multiplication.

One limitation of this method is that the tags *d*_*i*_ should be bounded by ciphertext dimension *N* to construct the encoding polynomial *DB*(*X*). Since the dimension *N* has a significant influence on the performance of HE scheme, too large value of *N* has an impractical impact on the performance. In the next section, we will describe how to overcome this problem in terms of the application to genomic data.

### Secure searching of biomarkers

We return to our main goal of task3: secure outsourcing matching of a set of biomakers to encrypted genomes. We describe how to encode and encrypt the genotype information of VCF file in order to apply the privacy-preserving database searching and extraction.

VCF file contains multiple genotype information lines, where each of them consists of a triple (*ch*_*i*_,*pos*_*i*_, *SNPs*_*i*_) of chromosome number, position, and a sequence of SNP alleles. A chromosome identifier *ch* ranges from 1 to 22, X, and Y. A non-negative integer *pos* represents the reference position with the first base having position 1, and *SNPs* is a reference or alternate sequence in {*A*,*T*,*G*,*C*}^∗^. A query from user is also a triple of the same form and we aim to decide absence/presence of this biomarker in the database file.

We represent the sex chromosomes X and Y as 0 and 23, respectively. Then we define an encoding function $\mathcal {E}: \mathbb {Z} \times \mathbb {Z} \rightarrow \mathbb {Z}$ by

$$(\mathsf{ch}, \mathsf{pos})\mapsto d = \mathsf{ch}+ 24 \cdot \mathsf{pos}. $$

In the following, we describe how to encode the *SNPs*. For convenience we set the upper bound for the length of *SNPs*, so let *n*_*SNP*_ be the maximal number of reference (or alternate) alleles to be compared between the query genome and user genome in the target database. Each of SNP is represented by two bits as

$$A\mapsto 00, \ T\mapsto 01, \ G\mapsto 10, \ C\mapsto 11, $$

and then concatenated with each other. Next we pad with 1 to the left of the bit string in order to express the staring position of SNPs. Finally it is zero-padded into a binary string of length *ℓ*_*SNP*_=2·*n*_*SNP*_+1, and we convert it into an integer value, denoted by *α*_*i*_. If a single nucleotide variant at the given locus is not known, then it is encoded as 0-string. For example, ‘*GC*’ is encoded as a bit string 1|10|11, which will be represented as an integer 1|10|11_(2)_=27.

Now consider the case that we wish to encode the reference and alternate alleles together. Let $\alpha _{i}^{\mathsf {ref}}$ and $\alpha _{i}^{\mathsf {alt}}$ denote the integer encodings of *n*_*SNP*_ reference alleles and *n*_*SNP*_ alternate alleles, respectively. Then we define an encoding *α*_*i*_ by the concatenation of two encodings, *i.e.,* $\alpha _{i}= 2^{\ell _{\mathsf {SNP}}} \cdot \alpha _{i}^{\mathsf {ref}}+ \alpha _{i}^{\mathsf {alt}}$ as an integer. Table 1 shows the format of database file and illustrates some examples of encoded genomic data.

**Table 1:** The format of genome data and its encoding with *n*_*SNP*_=10

*CHROM*	*POS*	*d*	*REF*	*ALT*	*α*
1	161235340	3869648161	*G*	*A*	12582916
1	161235596	3869654305	*C*	*T*	14680069
1	161235657	3869655769	*G*	*T*	12582917
1	161235981	3869663545	*G*	*A*	12582916
1	161237503	3869700073	·	*TTTTTGT*	21849
1	161237891	3869709385	*G*	*A*	12582916
1	161238009	3869712217	*G*	·	12582912
1	161238488	3869723713	*A*	*G*	8388614
1	161238683	3869728393	*G*	*A*	12582916
1	161238856	3869732545	*T*	·	10485760
1	161239028	3869736673	*AG*	·	37748736
1	161239142	3869739409	*A*	*G*	8388614
1	161239346	3869744305	*G*	*T*	12582917
1	161239470	3869747281	*C*	*T*	14680069
1	161239788	3869754913	·	*AA*	16
1	161239978	3869759473	*C*	*T*	12582917
1	161240641	3869775385	*TGAT*	·	740294656

A database file is encoded as a set of pair (*d*_*i*_,*α*_*i*_) for *i*=1,…,*n* such that $d_{i}= \mathcal {E}(\mathsf {ch}_{i},\mathsf {pos}_{i})$ and *α*_*i*_ is the encoded integer of the *i*-th SNP allele string. Then the encodings *d*_*i*_ and *α*_*i*_ are regarded as data-tag and value attribute, respectively. The data user constructs a polynomial $\mathsf {DB}(X)= \sum _{k} c_{k} X^{k}$ such that

$$c_{k}= \left\{\begin{array}{ll} \alpha_{i} & \text{if} \ k=d_{i} \ \text{for some} \ i,\\ \alpha \leftarrow \mathbb{Z}_{t}& \text{otherwise.} \end{array}\right. $$

The user encrypts the polynomial with the *RLWE* public-key encryption scheme as described above.

The query genes are also encoded as a pair of integers (*d*,*α*), however, we consider only the information of *d* is encrypted using the *RGSW* symmetric encryption scheme, that is, the user encrypts the monomial *X* ^−*d*^.

## Results and discussion

In this section, we explain how to set the parameters and describe our optimization techniques for the implementation. We also present our results using the techniques. The dataset was randomly selected from Personal Genome Project. Our implementation is publicly available on *github* [18].

### How to set parameters

Since all the matching computation is performed on encrypted data in the cloud, the security against a semi-honest adversary follows from the semantic security of the underlying HE scheme. The security of the homomorphic encryption scheme relies on the hardness of the *RLWE* assumption. We derive a lower-bound on the ring dimension as $N \geq \frac {\lambda +110}{7.2}\cdot \log _{2} Q$ to get *λ*-bit security level from the security analysis of [11].

Given the ciphertext modulus *Q*, it follows from the estimation of noise growth during evaluations [12] and decryption condition that we get the upper bound on the plaintext modulus *t* to ensure the correctness of decryption after computation. So we set *t* as the largest power-of-two integer less than the upper bound. If the encodings of the allele strings are too large, we divide them into smaller integers so that each of them is smaller than *t*. Then we repeat the algorithm to construct the corresponding polynomials of each integer.

### Optimization techniques

As we mentioned before, the ring dimension *N* needs to be larger than the encoded integers *d*_*i*_’s. However, the encoded integers *d*_*i*_ from VCF files have bits size about 32, while a dimension *N* with about 11≤ log2*N*≤16 is considered appropriate for implementation of HE schemes to achieve both security and efficiency. Hence direct application of our method to the VCF file would yield an impractical result.

For compression of tag data and its re-randomization, we make the use of a pseudo random number generator *H*(·) which transforms a tag *d*_*i*_ into a pair of two non-negative integers $d^{*}_{i}$ and $d^{\dagger }_{i}$ less than *N*. Our implementation adopts SHA-3 and extracts log2*N*=11 bits of the hashed value for each of $d^{*}_{i}$ and $d^{\dagger }_{i}$.



We construct two polynomials

$$\mathsf{DB}^{*}(X)= \sum_{k} c^{*}_{k} X^{k}, \ \mathsf{DB}^{\dagger}(X)= \sum_{k} c^{\dagger}_{k} X^{k} $$

by the Algorithm 2. Note that for any 1≤*i*≤*n* and $H(d_{i})=(d_{i}^{*},d_{i}^{\dagger })\in \{0,\dots,N-1\}^{2}$, the pair of constructed polynomials *DB* ^∗^ and *DB* ^*†*^ satisfy $\alpha _{i} = c_{d_{i}^{*}} + c_{d_{i}^{\dagger }}$. The procedure of database encoding for secure search of biomarkers is described in Algorithm 2.

Let $\mathsf {ct}^{*}_{\mathsf {DB}}$ and $\mathsf {ct}^{\dagger }_{\mathsf {DB}}$ denote the ciphertexts of the polynomials *DB* ^∗^ and *DB* ^*†*^, respectively. Similarly, given the query encoding *d*, the user computes its randomized value *H*(*d*)=(*d* ^∗^,*d* ^*†*^) and encrypts the two polynomials $X^{-d^{*}}$ and $X^{-d^{\dagger }}$. We denote the ciphertexts by $\mathsf {CT}^{*}_{\mathsf {Q}}$ and $\mathsf {CT}^{\dagger }_{\mathsf {Q}}$. The server computes the hybrid multiplication to obtain the ciphertexts

$$\mathsf{ct}^{*}_{\mathsf{mult}} = \mathsf{CT}^{*}_{\mathsf{Q}} \boxdot \mathsf{ct}^{*}_{\mathsf{DB}}, \ \mathsf{ct}^{\dagger}_{\mathsf{mult}} = \mathsf{CT}^{\dagger}_{\mathsf{Q}} \boxdot \mathsf{ct}^{\dagger}_{\mathsf{DB}}. $$

Now let *ct* denote the ciphertext computed by the homomorphic addition between $\mathsf {ct}^{*}_{\mathsf {mult}}$ and $\mathsf {ct}^{\dagger }_{\mathsf {mult}}$. Finally the server converts it into an *LWE* ciphertext and performs the modulus-switching procedure as described above. The Algorithm 3 describes the procedure of secure *search-and-extraction* using our proposed optimization techniques.



### Implementation results

The use of variable type ‘*int32 _t*’ accelerates the speed of implementations and basic C++ std libraries, so we set *Q*=2^32^ as the ciphertext modulus. We also set *t*=2^11^ as the modulus parameter of the plaintext space to ensure the correctness for the output ciphertext. We take the following parameters for Gadget matrix **G**: *B*_g_=128 and *d*_g_=5, so that they satisfy the condition ${B}_{\mathsf {g}}^{{{d}_{\mathsf {g}}}} \geq Q$.

Each coefficient of the secret key *sk* is chosen at random from {0,±1} and we set 64 as the number of nonzero coefficients in the secret key. As in the work of [12], we considered the Gaussian distribution of standard deviation *σ*=1.4 to sample random error polynomials.

For the efficiency of homomorphic multiplication, we also used the optimized library for complex FFT, *i.e.,* *the Fast Fourier Transform in the West* [19]. That is, we use the complex primitive 2*N*-th root of unity rather than a primitive root in a prime field of order *Q*. We measure a running time of 0.804 s to set up the FFT environment at dimension 2*N*=2^12^. The key generation of two schemes takes about 0.247 ms in total.

Table 2 presents the time complexity and storage for the evaluation of secure searching of biomarkers. All the experiments were performed on a single Intel Core i5 running at 2.9 GHz processor. The chosen parameters provide *λ*=128 bits of security level.

**Table 2:** Implementation results of secure searching of biomarkers

DB size	*n*_*SNP*_	Complexity				Storage		
		*Query*- *enc*	*DB*- *enc*	*Eval*	*Dec*	Query	DB	Result
10 K	2	3.247 ms	3.563 ms	0.018 s	0.004 ms	160 KB	3 MB	0.75 MB
	5		7.212 ms	0.039 s	0.011 ms		6 MB	1.5 MB
	10		14.813 ms	0.079 s	0.027 ms		12 MB	3 MB
100 K	2		21.424 ms	0.111 s	0.034 ms		17 MB	4.25 MB
	5		42.415 ms	0.227 s	0.064 ms		34 MB	8.5 MB
	10		99.921 ms	0.454 s	0.139 ms		68 MB	17 MB
4 M	2		0.745 s	3.954 s	1.171 ms		593 MB	148 MB
	5		1.506 s	7.911 s	1.949 ms		1185 MB	296 MB
	10		3.001 s	15.442 s	3.795 ms		2370 MB	593 MB

## Conclusions

In this work, we suggested an efficient method to securely search the query tag and extract the corresponding value from a database over hybrid *GSW* homomorphic encryption scheme. We came up with a solution to the secure outsourcing matching problem by using polynomial encoding and extraction of desired value based on the multiplication of an *RGSW* ciphertext and an ordinary *RLWE* ciphertext. And then we applied this method to find a set of biomarkers in DNA sequences.

Our solution shows the progress of cryptographic techniques in terms of their capability can support real-world genome data analysis in a cloud environment. We list a few fascinating open problems to remain. First, we only considered the semi-honest adversary model in this work. Other tools such as homomorphic authenticated scheme may lead to more efficient protocols in the malicious settings. Another issue is to support *k* multiple queries while maintaining the performance and communication cost less than *k* times of a single query case. We expect to have much faster performance by enabling a batching method.
